# Clinicopathologic Features and Outcomes in Fibronectin Glomerulopathy: A Case Series of 19 Patients

**DOI:** 10.3389/fmed.2020.00439

**Published:** 2020-08-14

**Authors:** Ti Zhang, Wei Zhang, Ke Zuo, Zhen Cheng

**Affiliations:** ^1^National Clinical Research Center of Kidney Diseases, Jinling Hospital, Nanjing University School of Medicine, Nanjing, China; ^2^Department of Urology, Changhai Hospital, Naval Medical University, Shanghai, China

**Keywords:** fibronectin glomerulopathy, outcome, prognosis, renal pathology, proteinuria

## Abstract

**Aims:** To describe the characteristics and prognosis of 19 patients with fibronectin glomerulopathy (FNG) and evaluate prognostic factors associated with poor renal outcomes.

**Methods:** Included in this retrospective study was 19 FNG patients in Nanjing Glomerulonephritis Registry system. Associations between the clinical parameters, pathological features, and renal outcomes were evaluated by Kaplan-Meier survival analysis.

**Results:** Of the 19 FNG patients included in this study, 8 (42.1%) were women. The median age of the 19 FNG patients was 31 (17–71) years, and the median disease duration 48 (1–175) months at diagnosis. At the time of renal biopsy, the mean serum creatinine (Scr) was 1.22 ± 0.16 mg/dl and urinary protein was 6.24 ± 0.97 mg/24 h. Renal biopsy showed a lobular appearance with cellular mesangial nodules expanded by matrix in 14 cases. After a median follow-up period of 87 months (interquartile range 34–114.5 months), 8 FNG patients developed renal function decline, including 7 progressing into end-stage renal disease (ESRD) and 1 presenting with by a 2-fold-increase in Scr. Scr and proteinuria remained stable in the remaining 11 patients. Kaplan-Meier survival analysis showed that nephrotic range proteinuria (*P* = 0.022) and focal glomerular sclerosis (*P* = 0.028) were associated with renal function decline.

**Conclusions:** Nephrotic range proteinuria and focal glomerular sclerosis were associated with renal function decline during the follow-up period of the FNG patients in our series. FNG Patients at risk of renal function decline should be identified preferentially and given more progressive and effective therapies to prevent further disease progression.

## Introduction

Fibronectin glomerulopathy (FNG) is a rare autosomal dominant inherited renal disease with typical clinical features of proteinuria, microscopic hematuria, and hypertension. The diagnosis of FNG mainly depends on renal biopsy. Pathologically, FNG is featured by massive deposition of fibronectin in the mesangium and along capillary walls. Electron microscopically, fibronectin deposition is shown as finely granular or fibrillary substructures with randomly arranged 12–16-nm fibrils (1–3). Fibronectin 1 (FN1) gene mutation was detected in about 40% of patients and is believed to be responsible for the occurrence of the disease (4–6). However, the exact pathogenic mechanism of FNG is not fully understood and no specific treatment is currently available.

So far only about 75 cases have been reported in the literature, mainly in case reports and case series with small sample sizes (7). The disease course and outcome of these cases need to be fully described. Moreover, it is believed that FNG patients usually show slow progression to ESRDover 15–20 years (8). In the series reported by Strøm et al. (1) some patients progressed into dialysis in 2 years, while renal function of some patients remained stable in over 10 years, suggesting that the clinical symptoms and renal function decline were variable over time, even among family members of the same pedigree. We report herein a largest series of patients with biopsy-proven FNG with long-term follow-up periods at a single center. The aim of this retrospective study was to describe the clinico-biological characteristics, renal pathology, and renal outcome of such patients and screen prognostic factors associated with poor renal outcomes.

## Materials and Methods

### Patients Selection

Nineteen biopsy-proven FNG registered in Nanjing Glomerulonephritis Registry System from 2007 to 2018 were enrolled and reviewed. The baseline and follow-up data of these patients were obtained from the database of the said registry system. All follow-up data were updated to September 2019. This study was conducted in compliance with the Good Clinical Practice protocol and the Declaration of Helsinki principles, and approved by the institutional review board.

### Follow-Up Observations and Outcome Measures

Clinical, biological, and histological data were collected retrospectively from the medical records of the patients at the time of kidney biopsy and at last follow-up. Hypertension was defined by systolic blood pressure >140 mm Hg and/or diastolic blood pressure >90 mm Hg and/or use of antihypertensive medications. Renal parameters included serum creatinine(SCr), urine protein and the estimated glomerular filtration rate (eGFR) estimated using the four-variable Modification of Diet in Renal Disease (MDRD) formula. Proteinuria and nephrotic range proteinuria were defined by urine protein >0.3 and >3 g/d, respectively. Nephrotic syndrome was defined by proteinuria >3.5 g/d with albuminemia <30 g/l. End-stage renal disease (ESRD) was defined as eGFR <15 mL/min/1.73 m^2^, initiation of dialysis or transplantation. Renal function decline was classified as a 2-fold increase in Scr after biopsy, initiation of dialysis, transplantation, or death based on the last clinical visit or report available.

### Biopsy Processing

Kidney biopsy samples were processed for light and immunofluorescence microscopy according to standard techniques. Sections were systematically stained with Congo red and examined under polarized light. At light microscopy, presence of glomerular sclerosis, endocapillary and mesangial cell proliferation, extracapillary proliferation, tubular atrophy and interstitial fibrosis, and vascular lesions were evaluated. The presence of immune deposits was determined on the basis of the immunostaining for IgG, IgM, IgA, C3, C1q, kappa, lamda light chains performed at the time of renal biopsy. Poly-clonal rabbit antibody (DAKO, A0245) was used in staining of fibronectin. The renal biopsy samples were reviewed blindly and independently by two experts in renal pathology.

### Statistical Methods

Descriptive statistics included the mean ± standard deviation (SD) or median (and range) as appropriate for continuous variables, and frequency (percentage) for categorical variables. Group comparisons were made using Student's *t-*test, Chi–square test, or Fisher's exact test, as appropriate. Renal survival was analyzed by using Kaplan–Meier curves and the log-rank test. A *p* < 0.05 was considered to indicate statistical significance. Data analyses were performed using SPSS software version 18.0 (SPSS, Inc., Chicago, IL, USA).

## Results

### Patient Baseline Characteristics

The 19 patients (8 female and 11 male) with biopsy-proven FNG, including previously published ones, were identified in this study (9). The patient baseline characteristics are detailed in Table 1. The median age at diagnosis was 31 (range 17–71) years. The intervals between initial onset of renal disease and the date of renal biopsy varied (median 48 months, range 1–175). The family history of renal disease in 9 patients was listed in Table 1. The baseline median SCr was 1.07 mg/dl (range 0.6–3.13). Distribution of the patients by 2012 KDIGO CKD guideline (CKD evaluation and management) at baseline using the MDRD formula is as follows: 11 (57.9%) within G1A3, 5 (26.3%) within G2A3, 2 (10.5%) within G3aA3, and 1 (5.3%) within G3bA3. All patients had proteinuria with median 5.73 g/d (range 1.3–17.8) and no patient demonstrated gross hematuria. Nephrotic range proteinuria was detected in 14 patients (73.7%). Median serum albumin was 31.6 g/L (range 21.6–47.2). Full-blown nephrotic syndrome was observed in 6 patients (31.6%). Hypertension occurred in 13 patients (68.4%). Genetic tests were performed in 2 patients, including one with heterozygous missense mutation L1974P. No specific comorbidities including neoplasms or congenital alterations was detected in our series.

**Table 1:** Patient demographics and renal characteristics.

**Patients**	**Age**	**Gender**	**Follow-up time**	**Hypertension**	**Nephrotic syndrome**	**Creatinine at renal biopsy (mg/dl)**	**Proteinuria (g/24 h)**	**Serum albumin**	**Family history of renal disease**
1	65	Female	25	Yes	No	1.07	5.56	31.6	No
2	71	Male	71	Yes	Yes	1.13	6.99	27.5	Two sisters on dialysis
3	22	Male	40	Yes	No	1.55	2.49	39.1	One cousin diagnosed with MCD
4	31	Male	40	Yes	Yes	1,27	7.9	28.4	Mother with proteinuria
5	43	Female	34	No	No	0.6	1.5	36.5	Father on dialysis
6	29	Female	25	No	No	0.72	3.71	30.3	Father, one brother and one sister on dialysis
7	30	Male	113	Yes	No	0.78	5,73	35.9	Mother on dialysis, one sister diagnosed with FNG
8	34	Female	95	Yes	Yes	0.74	5.36	18.9	Mother on dialysis, one brother diagnosed with FNG
9	35	Male	87	No	No	1.83	6.02	32.1	Father, aunt and a cousin on dialysis
10	45	Female	140	No	No	0.72	1.44	39.7	No
11	24	Male	92	Yes	No	2.81	3.91	31.2	No
12	45	Male	140	Yes	No	0.9	6.85	39	A sister diagnosed with polycystic kidney disease
13	32	Male	147	No	No	0.83	1.33	47.2	No
14	27	Female	118	No	No	0.71	1.89	36.9	No
15	26	Male	109	Yes	Yes	1.37	8.2	22.4	No
16	19	Male	14	Yes	Yes	1.07	11.4	25.5	No
17	32	Female	116	Yes	Yes	3.31	9.13	21.6	No
18	29	Male	53	Yes	No	1.22	11.47	30.7	No
19	19	Female	24	No	No	0.59	17.78	36.3	No

*MCD, minimal change disease; FNG, fibronectin glomerulopathy*.

### Renal Biopsy Findings

The renal biopsy samples of all patients were available for review (Table 2). Lobular lesions (73.6%) were the predominant histologic pattern, characterized by mesangial expansion, lobular accentuation, positive periodic acid-Schiff staining, and fuchsinophilic deposits. Lobular accentuation of the glomerular tuft was observed in all cases. There was a range of 0–78.6% global glomerulosclerosis (mean 14.2%). Focal glomerular sclerosis was present in 5 cases (26.3%) with a mean of 8.1%. Interstitial fibrosis and tubular atrophy were reported in 15 cases (78.9%), including 5 cases of the moderate form. The acute form tubular necrosis was seen in 2 cases (10.5%) and moderate-to-severe form in one case (5.26%). Arteriolar hyalinosis and arterial intimal thickening were observed in 12 (63.1%) and 5 (26.1%) cases, respectively. Besides strong positive staining of fibronectin, immunofluorescence microscopy also showed slight and irregular deposition of IgM in 5 cases, IgG in 6, IgA in 7, C3 in 7 cases on the mesangial and capillary wall. Congo-red staining was negative in all cases. Electron microscopy showed massive granular deposits in the mesangial and subepithelial area.

**Table 2:** Renal biopsy findings.

**Patients**	**Lesion pattern**	**% Global glomerusclerosis**	**% Focal glomerular sclerosis**	**Interstitial fibrosis**	**Immunofluorescence microscopy**
1	Lobular	35.7	0	Mild	IgA (1+), C3 (1+)
2	Lobular	17.6	11.8	Mild	IgA (1+), IgG (1+), C3 (1+)
3	MPGN	25	0	Moderate	IgM (1+), C3 (1+)
4	Lobular	0	0	Mild	C3 (1+)
5	Lobular	2.7	0	Negative	Negative
6	Lobular	0	0	Mild	Negative
7	Lobular	10.2	5.13	Negative	IgG (1+), IgA (1+)
8	Lobular	7.7	3.8	Mild	IgM (1+), C3 (1+)
9	MPGN	20	0	Moderate	IgG (1+), IgA (1+), IgM (1+)
10	Lobular	3	0	Mild	Negative
11	Lobular	78.6	0	Moderate	IgG (1+), IgA (1+), IgM (1+)
12	MPGN	0	0	Mild	IgA (1+), IgG (1+)
13	Lobular	0	0	Negative	IgM (1+)
14	Lobular	0	0	Negative	Negative
15	Lobular	9.3	0	Mild	IgG (1+), IgA (1+), C3 (1+)
16	Lobular	20	5	Moderate	C3 (1+)
17	MPGN	25.7	0	Moderate	Negative
18	Lobular	12.5	15	Mild	Negative
19	MPGN	3.3	0	Mild	Negative

*MPGN, membranoproliferative glomerulonephritis*.

### Treatment and Renal Outcomes

Renal outcomes, treatment and follow-up findings of the patients are presented in Table 3. All the 19 patients were treated with renin-angiotensin system blockade, including 11 patients who were treated with Tripterygium Wilfordii Hook (TWHF), and 4 patients with corticosteroid therapy in combination with immunosuppressive therapies, including 2 with mycophenolate mofetil (MMF) and 2 with tacrolimus.

**Table 3:** Renal outcomes, treatment, and follow-up findings.

**Patients**	**F/U serum creatinine (mg/dl)**	**Dialysis at last FU**	**F/U proteinuria (g/24 h)**	**Duration of F/U in months**	**Duration of renal biopsy to dialysis in months**	**Treatment**
1	1.06	No	1.26	25		Prednisone/MMF/ARB
2	ND	Yes	ND	71	71	TWHF/ARB
3	1.53	No	0.68	40		TWHF/ARB
4	1.01	No	10.9	40		ARB
5	0.61	No	0.1	34		ARB
6	0.59	No	4.11	25		Prednisone/Tacrolimus/ARB
7	ND	Yes	ND	113	28	TWHF/ARB
8	ND	Yes	ND	95	90	TWHF/ARB
9	ND	Yes	ND	87	39	ARB
10	0.7	No	0.8	140		TWHF/ARB
11	ND	Yes	ND	92	92	Prednisone/TWHF/ARB
12	1.58	No	1.19	140		ARB
13	1.19	No	6.81	147		TWHF/ARB
14	0.85	No	2.31	118		Prednisone/Tacrolimus
15	ND	Yes	ND	109	109	TWHF/ARB
16	1.49	No	3.68	14		TWHF/ARB
17	ND	Yes	ND	116	20	TWHF/ARB
18	2.48	No	10.9	53		TWHF/ARB
19	0.57	No	2.77	24		Prednisone/MMF

*F/U, follow-up; MMF, mycophenolate mofetil; TW, Tripterygium wilfordii Hook F; ARB, angiotensin receptor blockers; ND, not done*.

Last follow-up data were available in 15 of the 19 patients. The mean follow-up duration was 78 months (rang 14–147 months, median 87). At last follow-up, 7 patients progressed to ESRD despite supportive therapy and required initiation of dialysis, 2 of whom received renal transplantation. Scr increased by 2-fold in one patient. Of the 8 patients, the mean SCr and mean proteinuria at time of renal biopsy was 1.62 ± 0.31 mg/dl and 7.10 ± 0.85 mg/24 h, respectively. The time from renal biopsy to the initiation of dialysis ranged from 21 months to over 109 months (median 71). The mean SCr in patients not on dialysis remained stable from 0.91 ± 0.09 mg/dl at the time of renal biopsy to 1.01 ± 0.11 mg/dl at the last follow-up and the mean proteinuria improved from 5.62 ± 1.56 mg/24 h to 3.14 ± 0.97 mg/24 h, respectively.

### Factors Associated With Renal Function Decline

**Figure 1:**
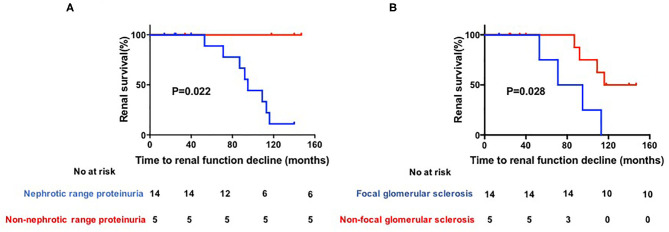
**(A,B)** Kaplan–Meier estimates of significant loss of renal function. Renal function was defined as impaired if serun creatinine (Scr) increased by 2-fold after biopsy, initiation of dialysis, transplantation or death.

The general characteristics, laboratory results, and histological characteristics of these patients were included for statistical analysis. The results showed that the median follow-up duration did not differ between patients with stable renal function and those with renal function decline [40 months [IQR 25–129 months] vs. 93.5 months [IQR 83–110 months]; *P* = 0.26]. Patients with nephrotic range proteinuria had a significantly higher probability of renal function decline than non-nephrotic range proteinuria patients [8 of 14 patients [57.1%] compared to 0 of 5 patients [0.0%]; *P* = 0.022]. Pathologically, patients with focal glomerular sclerosis also had a trend toward higher risk of renal function decline than non-focal glomerular sclerosis patients [4 of 5 patients [80.0%] compared to 4 of 14 patients [28.5%]; *P* = 0.028] (Figure 1).

## Discussion

In this study, we described the clinicopathologic features of FNG patients in a single center. To the best of our knowledge, this is the first study to have demonstrated the factors associated with poor renal outcomes in FNG patients.

Most FNG patients clinically present with mild proteinuria, microscopic haematuria and hypertension. In the 76 cases reported by Takii et al. (8), proteinuria was the most common presenting symptom in all cases, of which 35 out of 69 (50.7%) had nephrotic-range proteinuria. In our series, the proportion of nephrotic range proteinuria was 73.6%, suggesting that high-grade proteinuria is a common occurrence in Chinese FNG patients. It is generally accepted that high-grade proteinuria is positively correlated with increased risk of ESRD (10, 11). Indeed, nephrotic range proteinuria is also indicated as a poor renal prognostic factor in FNG patients in our series.

Also, 6 (31.5%) patients in our study presented with abnormal serum creatinine (> 1.24 mg/dl) and 4 (21.0%) with decreased renal function (GFR <60 ml/min) in our study. Among the 76 cases reported by Takii et al. (8) Scr or renal function was recorded in 58 patients. Abnormal Scr (> 1.24 mg/dl) or renal function (GFR <60 ml/min) was detected in 20 patients (34.4%), two of whom presented with ESRD at the time of diagnosis. The rate of renal function decline is similar to that exhibited in our study.

Typically, FNG is regarded as a slowly progressive glomerular disease into ESRD over the period of 15–20 years (2, 10). Most FNG patients progressed into ESRD in the 2nd to 6th decade of life. However, the period of progressing into ESRD varies based on previous studies. Strom et al. (1) reported that the mean time of 4 FNG patients progressing into dialysis was 93 months, while Gemperle et al. (12) reported 5 patients who needed dialysis with 160-month follow-up (1). In our series, the mean time of progression into ESRD or dialysis is 71 months, which may mean that the rate of disease progression in FNG used to be underestimated previously (13).

A genetic test should be performed when FNG is suspected pathologically (6, 14). In 2008, Castelletti et al. (5) firstly confirmed mutations at the FN1 gene locus at 2q32. They identified three heterozygous missense mutations Y973C, W1925R, and L1974R. Subsequently, Ohtsubo et al. (4) identified six FN1 mutations including five novel FN1 mutations. In our study, genetic test results were only available in 2 patients, including heterozygous missense mutation L1974P in one patient, and the mutation site in this patient is consistent with that reported by Castelletti et al. (5). However, none of these previously identified FN1 mutations was found in the other patient.

Deposition of immunoglobulins or complement components in the glomeruli is a commonly observed phenomenon in immunofluorescent study. Yoshino et al. (15) reported detection of immunoglobulin, fibrinogen, or complement components in 15 of their 43 cases (35%) (16). The link between immunoglobulin or complement component deposition and prognosis has not been clearly defined. In our study, we observed co-deposition of fibronectins with immunoglobulin or C3 in the mesangium in 12 (63.1%) of the 19 cases. Whether immunoglobulin or C3 deposition is non-specific or represents immune complex-mediated glomerulonephritis needs further investigation. Focal glomerular sclerosis as a typical histological change was rarely mentioned in FNG in previous studies. Indeed, focal glomerular sclerosis was only detected in five patients in our series and found to be associated with poor renal survival. Surprisingly, tubulointerstitial fibrosis or vascular changes was not found to be significantly correlated with the renal outcome.

Our study has several limitations. First, the population of enrolled FNG patients is relatively small and therefore the implications and outcomes should be interpreted with caution. Second, although our study provided a mean 10-years follow-up observation, loss to follow-up still occurred in some cases. Finally, as a result of the retrospective nature study, we were unable to evaluate the effect of treatment on outcomes. Because treatment of FNG is always individualized, it is therefore difficult to accurately evaluate the influence of treatment on renal outcomes.

In conclusion, the disease progression of FNG may be underestimated, which should not be ignored in some progressive renal diseases. In our series, nephrotic range proteinuria and focal glomerular sclerosis were found to be associated with renal function decline in FNG patients during follow-up. It will help clinicians to identify patients at risk of renal progression in clinical practice. Further studies regarding to underlying mechanism of FNG and finding effective treatments are necessary.

## Data Availability Statement

The raw data supporting the conclusions of this article will be made available by the authors, without undue reservation.

## Author Contributions

TZ and ZC: research conception and study design and research and data analysis. TZ and WZ: manuscript preparation and clinical data collection. TZ and KZ: research sample preparation. All authors have read the journal's authorship agreement and agree with the statements. Each author contributed important intellectual content during manuscript drafting or revision and accepts accountability for the overall work by ensuring that questions pertaining to the accuracy or integrity of any portion of the work are appropriately investigated and resolved.

## Conflict of Interest

The authors declare that the research was conducted in the absence of any commercial or financial relationships that could be construed as a potential conflict of interest.
